# Supplementary material for: Quasi-Movements and “Quasi-Quasi-Movements”: Does Residual Muscle Activation Matter?
Source: Life (Basel). 2023 Jan 21;13(2):303. doi: 10.3390/life13020303 (PMC9964598; doi:10.3390/life13020303)
Supplement: Supplementary file 1 [file life-13-00303-s001.zip › life-2075141-supplementary.pdf]

Supplementary Materials

Quasi-movements and “quasi-quasi-movements”: does residual muscle activation matter? by Anatoly N. Vasilyev, Artem S. Yashin, Sergei L. Shishkin

A

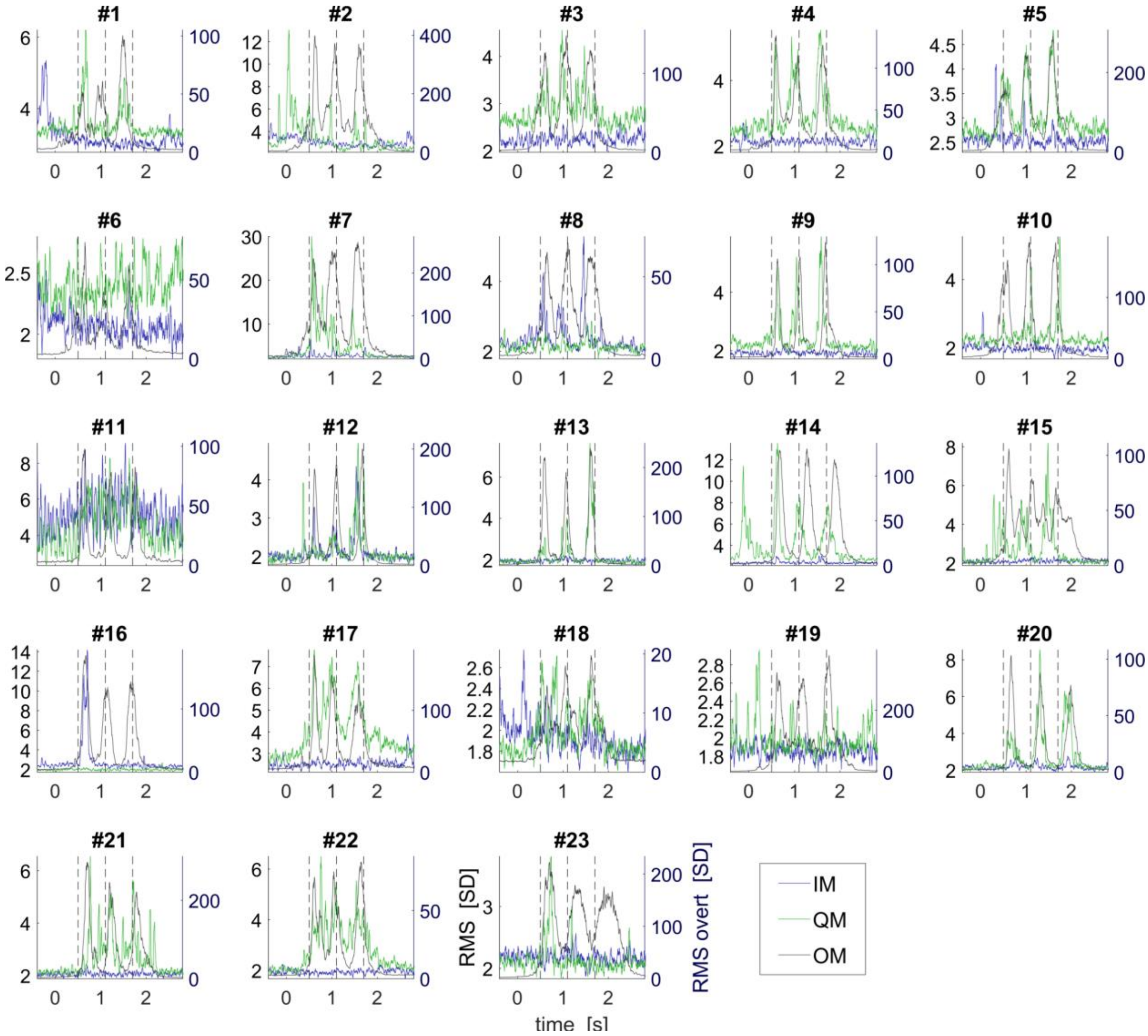

B

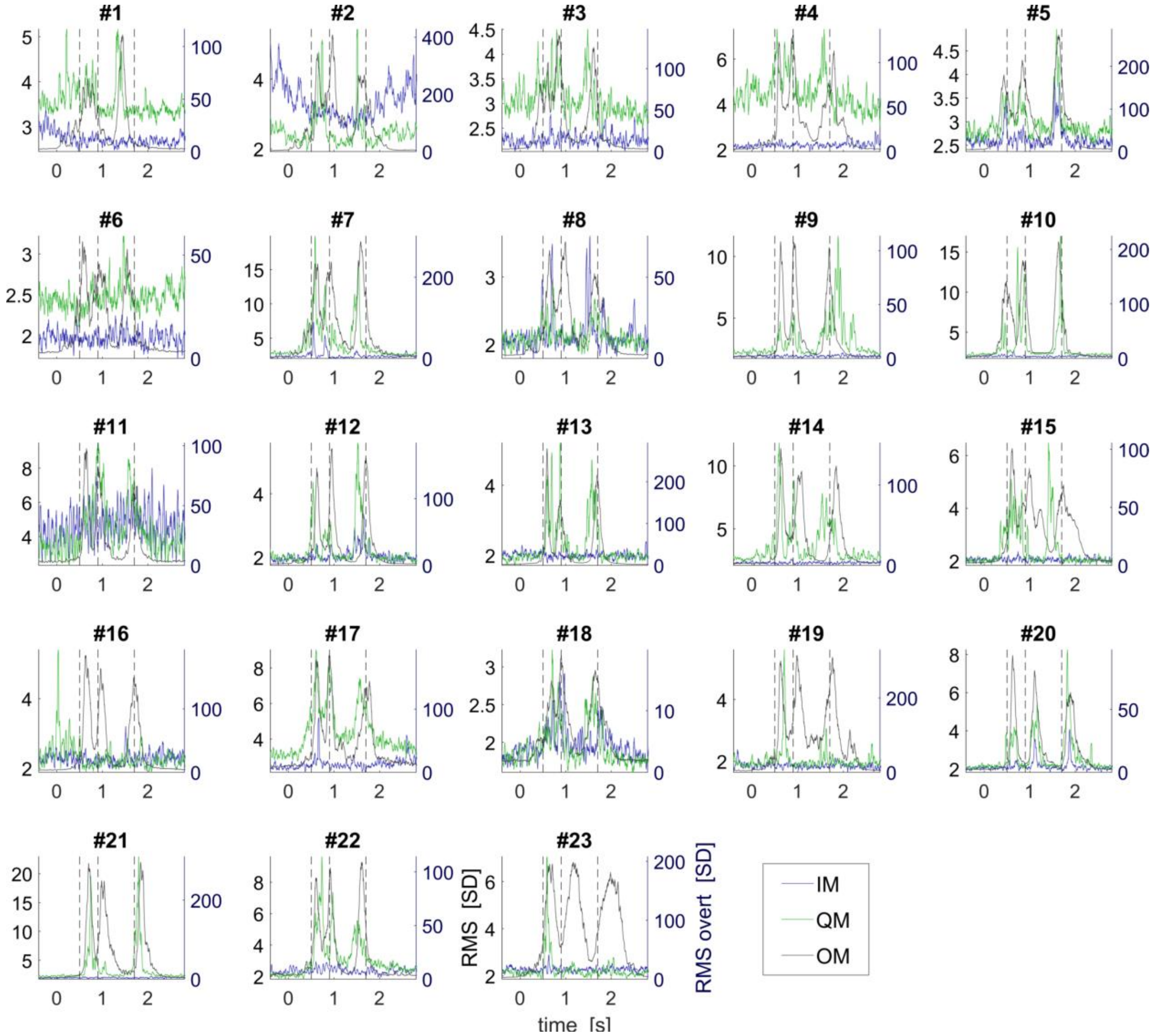

Figure S1. Individually averaged EMG RMS time courses for three conditions: OM, overt movements; QM, quasi-movements and IM, imaginary movements, and periodic (A) and aperiodic (B) rhythmic patterns.

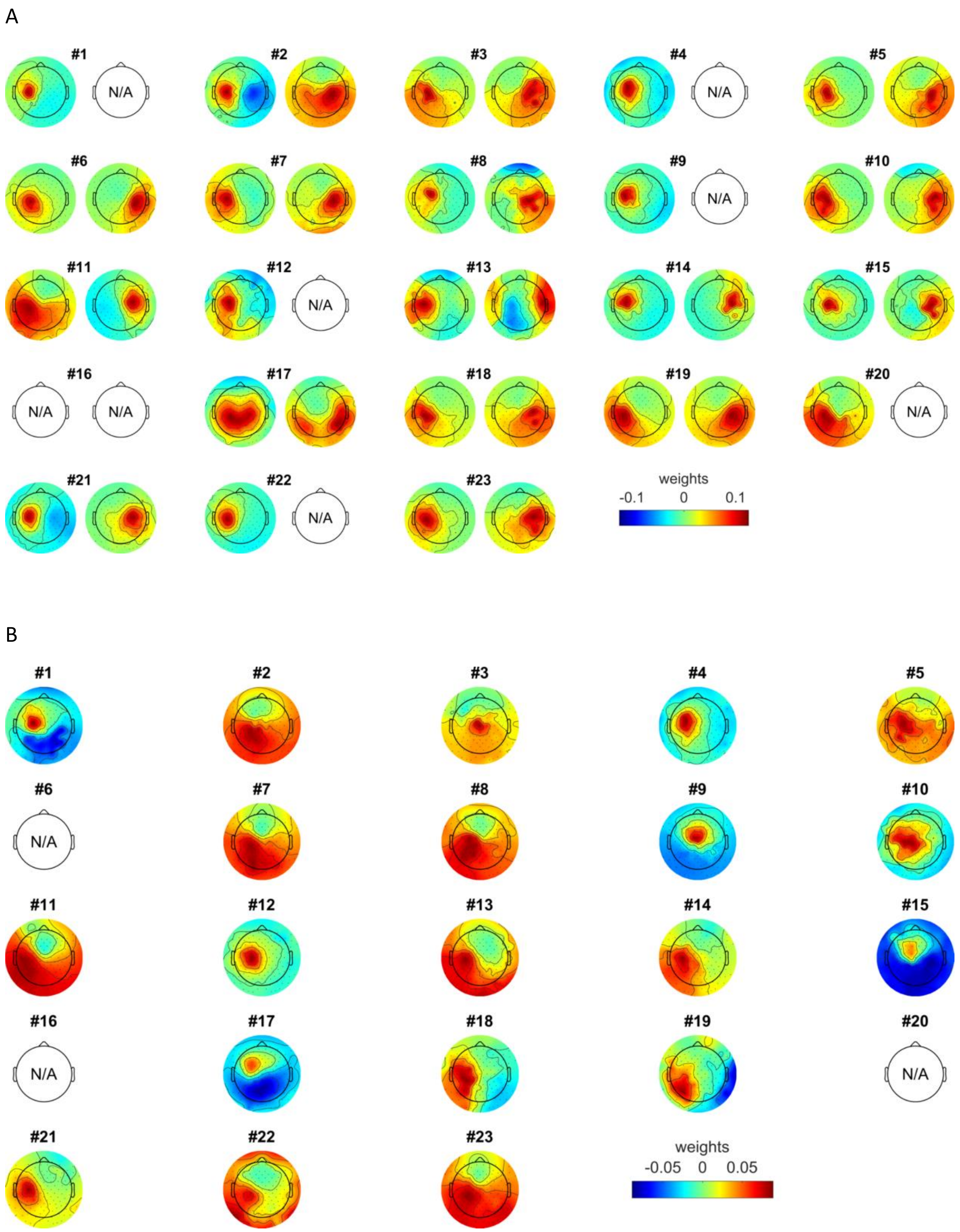

Figure S2: Scalp topographies for movement-sensitive EEG components: (A) contralateral ERD (alpha band), ipsilateral ERD (alpha band) (left and right head in each pair) and (B) contralateral ERS (beta band). The topographies represent the weights of selected columns in each participant's activation matrix A (for details see step 10 of the pipeline described in "Setting individual frequency bands and spatial filters" subsection of Methods). Non-colored heads are shown where no corresponding component was found.

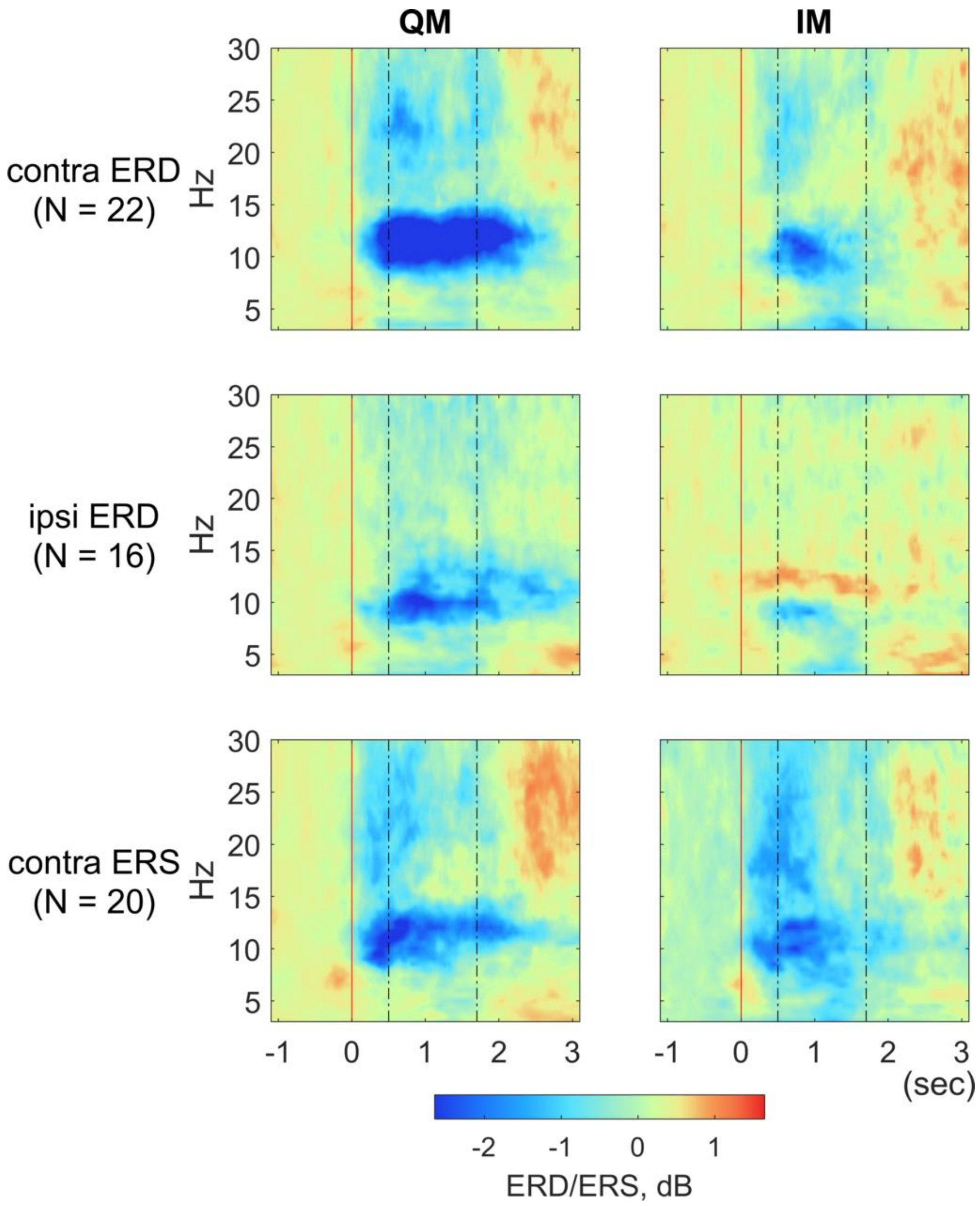

Figure S3: Grand average for the EEG component spectrograms. The spectrograms were obtained by applying the Morlet wavelet with varying number of cycles (“superlets”, [10]) to the EEG sensorimotor components (*contra ERD, ipsi ERD, contra ERS*) from the covert movement conditions (QM, IM). 0 ms corresponds to the visual cue onset. Power values were converted into dB with normalizing by the average over a time interval preceding the visual cue (-1000..-450 ms). Vertical lines show the onsets of the sound.

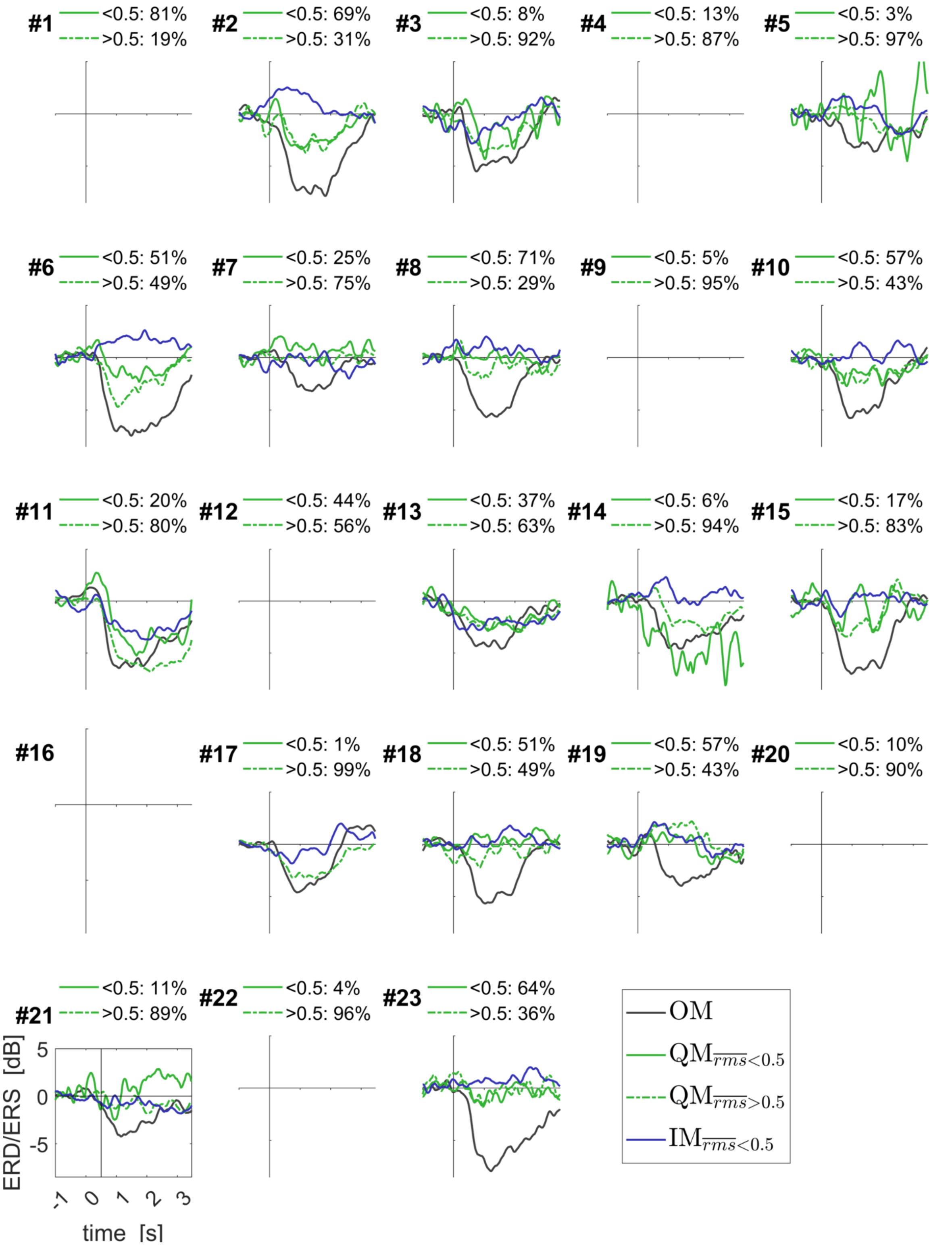

Figure S4: Individually averaged time courses of the ERD/ERS for the ipsilateral alpha band component of the EEG sensorimotor rhythms. Data for periodic and aperiodic sound rhythms were put together. Individual percentages of trials are shown for QM with low and elevated EMG RMS, as quantified by $\underline{rms}$. See the capture of Figure 7 for more details, Figure 8 and S5 for other components.

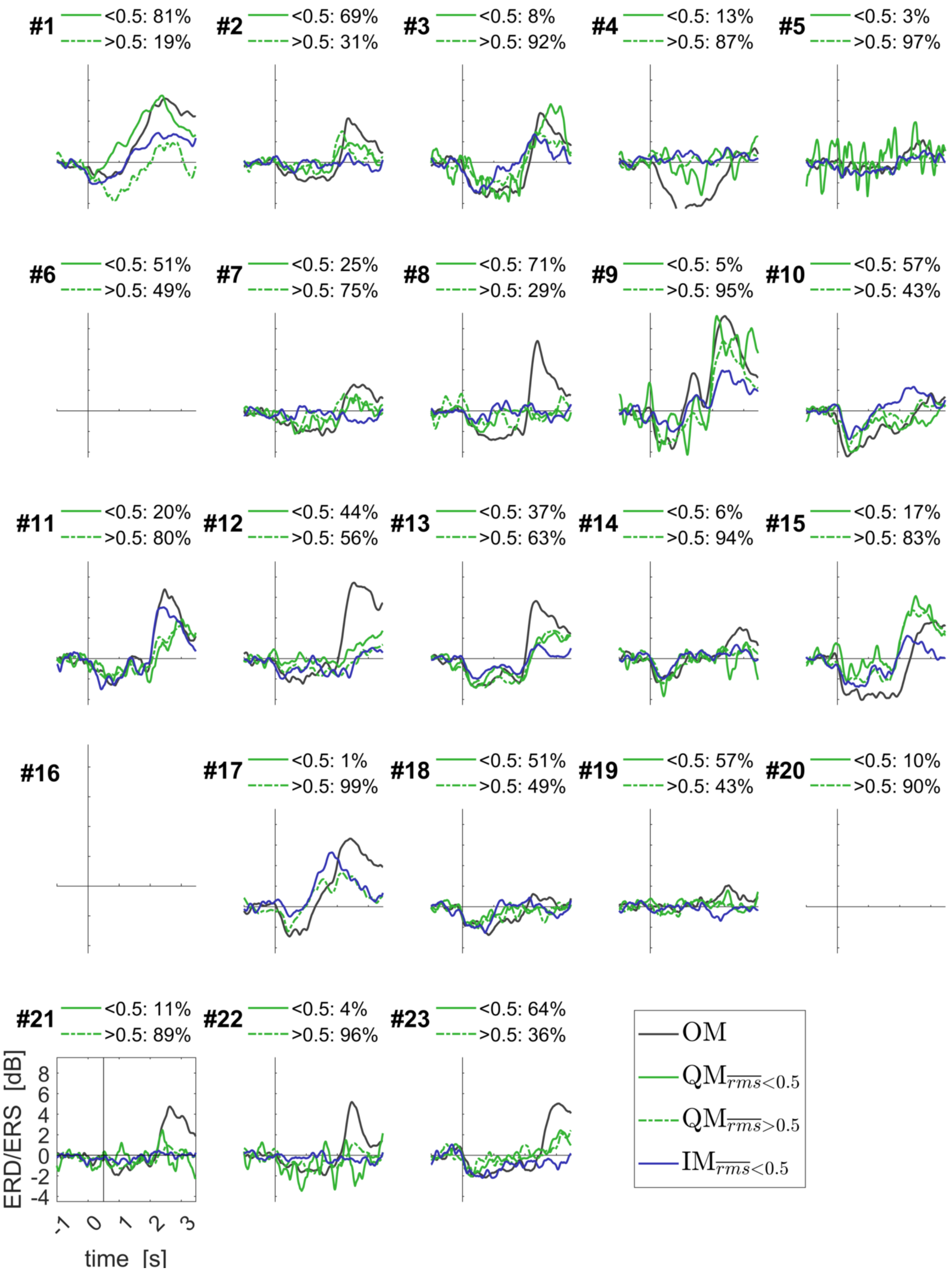

Figure S5: Individually averaged time courses of the ERD/ERS for the contralateral beta band component of the EEG sensorimotor rhythms. Data for periodic and aperiodic sound rhythms were put together. Individual percentages of trials are shown for QM with low and elevated EMG RMS, as quantified by $\underline{rms}$. See Figure 7 capture for more details, Figure 8 and S4 for other components.

Table S1: Individual movement-reactive frequency bands for the ERD (alpha band) and ERS (beta band) components of the EEG sensorimotor rhythm.

Participant	Alpha band, Hz		Beta band, Hz	
1	10	13	13	22
2	11	14	15	28
3	10	13	23	30
4	10.5	13.5	17	27
5	10	13	25	32
6	12.5	15.5	-	-
7	10.5	13.5	15	25
8	10.5	13.5	15	23
9	10	13	15	23
10	11	14	15	22
11	9	12	23	30
12	9	12	15	23
13	9	12	13	25
14	12.5	15.5	18	27
15	11	14	15	21
16	-	-	-	-
17	8	11	15	23
18	10	13	15	21
19	11	14	15	22
20	10	13	-	-
21	12.5	15.5	18	31
22	9.5	12.5	15	22
23	11.5	14.5	16	22
Mean±SEM	10.41±0.25	13.41±0.25	16.55±0.75	24.95±0.8

Table S2: LMM results for the contralateral alpha band ERD component of the EEG sensorimotor rhythms. Prefixes in '*cond_quasi', 'rhythm_periodic'* indicate that coefficients were estimated for the levels '*cond'* = 'quasi', '*rhythm'* = 'periodic'.

Name	*Estimate*	*SE*	*tStat*	*DF*	*pValue*	*Lower*	*Upper*
'(Intercept)'	0,05481	0,05724	0,9575	3587	0,33838	-0,05742	0,16704
'cond_quasi'	**-0,36010**	**0,05988**	**-6,0141**	**3587**	**0,00000**	**-0,47750**	**-0,24271**
'rhythm_periodic'	-0,02266	0,03217	-0,7043	3587	0,48131	-0,08574	0,04042
'recN'	*0,01105*	*0,00558*	*1,9800*	*3587*	*0,04778*	*0,00011*	*0,02200*
'trialN'	*0,01133*	*0,00570*	*1,9862*	*3587*	*0,04708*	*0,00015*	*0,02251*
'prevT'	-0,00026	0,03265	-0,0081	3587	0,99354	-0,06427	0,06374
'peakRMS'	-0,04110	0,04385	-0,9373	3587	0,34866	-0,12708	0,04487
'prePower'	**0,15160**	**0,01620**	**9,3609**	**3587**	**0,00000**	**0,11985**	**0,18335**
'cond_quasi:peakRMS'	0,06248	0,04969	1,2574	3587	0,20870	-0,03494	0,15990

Table S3: LMM results for the ipsilateral alpha band ERD component of the EEG sensorimotor rhythms. Prefixes in '*cond_quasi', 'rhythm_periodic'* indicate that coefficients were estimated for the levels '*cond'* = 'quasi', '*rhythm'* = 'periodic'.

Name	*Estimate*	*SE*	*tStat*	*DF*	*pValue*	*Lower*	*Upper*
'(Intercept)'	0,04588	0,06204	0,7395	2643	0,45965	-0,07578	0,16754
'cond_quasi'	**-0,28407**	**0,04321**	**-6,5739**	**2643**	**0,00000**	**-0,36880**	**-0,19933**
'rhythm_periodic'	-0,01463	0,03708	-0,3946	2643	0,69320	-0,08735	0,05808
'recN'	0,00944	0,00642	1,4707	2643	0,14149	-0,00314	0,02202
'trialN'	0,00109	0,00658	0,1650	2643	0,86896	-0,01181	0,01398
'prevT'	0,07615	0,03760	2,0254	2643	0,04293	0,00243	0,14987
'peakRMS'	**-0,09292**	**0,04044**	**-2,2979**	**2643**	**0,02165**	**-0,17221**	**-0,01363**
'prePower'	**0,21475**	**0,01861**	**11,5400**	**2643**	**0,00000**	**0,17826**	**0,25124**
'cond_quasi:peakRMS'	0,01393	0,04622	0,3014	2643	0,76312	-0,07670	0,10456

Table S4: LMM results for the contralateral beta band ERS component of the EEG sensorimotor rhythms. Prefixes in '*cond_quasi', 'rhythm_periodic'* indicate that coefficients were estimated for the levels '*cond'* = 'quasi', '*rhythm'* = 'periodic'.

Name	*Estimate*	*SE*	*tStat*	*DF*	*pValue*	*Lower*	*Upper*
'(Intercept)'	**-0,52258**	**0,05899**	**-8,8590**	**3191**	**0,00000**	**-0,63824**	**-0,40692**
'cond_quasi'	**0,25387**	**0,07109**	**3,5713**	**3191**	**0,00036**	**0,11449**	**0,39325**
'rhythm_periodic'	0,00510	0,03322	0,1535	3191	0,87799	-0,06003	0,07023
'recN'	**0,03257**	**0,00575**	**5,6646**	**3191**	**0,00000**	**0,02130**	**0,04384**
'trialN'	**0,03197**	**0,00589**	**5,4301**	**3191**	**0,00000**	**0,02043**	**0,04352**
'prevT'	-0,05483	0,03366	-1,6287	3191	0,10347	-0,12083	0,01117
'peakRMS'	**0,16303**	**0,04600**	**3,5442**	**3191**	**0,00040**	**0,07284**	**0,25322**
'prePower'	**0,04863**	**0,01666**	**2,9187**	**3191**	**0,00354**	**0,01596**	**0,08130**
'cond_quasi:peakRMS'	**-0,12727**	**0,05221**	**-2,4376**	**3191**	**0,01484**	**-0,22965**	**-0,02490**
